# A Connectome-Based Comparison of Diffusion MRI Schemes

**DOI:** 10.1371/journal.pone.0075061

**Published:** 2013-09-20

**Authors:** Xavier Gigandet, Alessandra Griffa, Tobias Kober, Alessandro Daducci, Guillaume Gilbert, Alan Connelly, Patric Hagmann, Reto Meuli, Jean-Philippe Thiran, Gunnar Krueger

**Affiliations:** 1 Signal Processing Laboratories (LTS5), Ecole Polytechnique Fédérale de Lausanne, Lausanne, Switzerland; 2 Advanced Clinical Imaging Technology, Siemens Schweiz AG-CIBM, Lausanne, Switzerland; 3 Department of Radiology, Centre Hospitalier de l’Université de Montréal, Montreal, Quebec, Canada; 4 Brain Research Institute, Florey Neuroscience Institutes (Austin), Melbourne, Australia; 5 Department of Medicine, University of Melbourne, Melbourne, Australia; 6 Department of Radiology, Centre Hospitalier Universitaire Vaudois and University of Lausanne, Lausanne, Switzerland; University of Alberta, Canada

## Abstract

Diffusion MRI has evolved towards an important clinical diagnostic and research tool. Though clinical routine is using mainly diffusion weighted and tensor imaging approaches, Q-ball imaging and diffusion spectrum imaging techniques have become more widely available. They are frequently used in research-oriented investigations in particular those aiming at measuring brain network connectivity. In this work, we aim at assessing the dependency of connectivity measurements on various diffusion encoding schemes in combination with appropriate data modeling. We process and compare the structural connection matrices computed from several diffusion encoding schemes, including diffusion tensor imaging, q-ball imaging and high angular resolution schemes, such as diffusion spectrum imaging with a publically available processing pipeline for data reconstruction, tracking and visualization of diffusion MR imaging. The results indicate that the high angular resolution schemes maximize the number of obtained connections when applying identical processing strategies to the different diffusion schemes. Compared to the conventional diffusion tensor imaging, the added connectivity is mainly found for pathways in the 50–100mm range, corresponding to neighboring association fibers and long-range associative, striatal and commissural fiber pathways. The analysis of the major associative fiber tracts of the brain reveals striking differences between the applied diffusion schemes. More complex data modeling techniques (beyond tensor model) are recommended 1) if the tracts of interest run through large fiber crossings such as the centrum semi-ovale, or 2) if non-dominant fiber populations, e.g. the neighboring association fibers are the subject of investigation. An important finding of the study is that since the ground truth sensitivity and specificity is not known, the comparability between results arising from different strategies in data reconstruction and/or tracking becomes implausible to understand.

## Introduction

Over recent years, there has been a growing interest in investigating the connectivity profile of the entire brain, referred to by the scientific community as the human connectome [1], [2]. By allowing *in vivo* imaging of the brain’s major fiber pathways, diffusion MR tractography [3]–[6] has turned out to be a promising technique to map the connectome at the millimeter scale. Recently, several groups have independently proposed to build structural connection matrices from diffusion MR tractography using various diffusion acquisition protocols and models [7]–[10].

Diffusion Tensor Imaging (DTI) is a frequently-used method to model the diffusion data in order to obtain orientational information. This method maps the orientation of fibers by fitting a second-order symmetric tensor on the diffusion data [11]. However, the use of a single tensor limits DTI to a single direction of maximum diffusion inside each imaging voxel. Consequently, DTI fails to correctly map diffusion in voxels where two or more fiber populations interfere [12]. Behrens et al. concluded that one third of white matter voxels may be affected by this problem [13], and later work by Jeurissen et al. reported finding two or more fiber orientations in 90% of white matter voxels [14].

Other approaches have been proposed to address this issue, such as Diffusion Spectrum Imaging (DSI), which allows measuring the diffusion spectrum [15]–[17]. This method requires specific diffusion encoding schemes, by which three-dimensional **q**-space is sampled, usually following a Cartesian grid. DSI was validated with phantoms made of parallel capillaries filled with water, as well as with manganese-enhanced rat optic tracts. The results showed that the crossing fiber orientations estimated with DSI were in excellent agreement with the results from histology [18], [19].

Subsequently, another diffusion acquisition has emerged, called high angular resolution diffusion imaging (HARDI). These acquisitions are characterized by a large number of diffusion encoding gradients distributed over a single shell in **q**-space. Several reconstruction schemes can be used to analyze HARDI data, such as Q-Ball Imaging (QBI) [20] or Constrained Spherical Deconvolution (CSD) [21]. QBI is a model-free reconstruction scheme measuring the angular structure of the diffusion spectrum. It has been shown to allow the mapping of complex diffusion structures in areas of *crossing* and *kissing* fibers, such as the intersection between the optic radiation and the splenium of the corpus callosum, Meyer's loop, or the middle temporal gyrus [22].

Though HARDI-based approaches and DSI have undoubtedly the potential to better disentangle complex fiber structures compared to DTI, it still remains unclear to which level these techniques provide a gain for *in vivo* whole-brain tractography and thus in clinical research, nor whether respective scalar maps may provide additional information over the fractional anisotropy (FA) or apparent diffusion coefficient (ADC) maps.

Behrens et al. suggested that higher order reconstruction schemes should increase the sensitivity of tractography as compared to DTI [13], especially for non-dominant fiber tracts. More recently, Wedeen et al. made a comparison between DSI and DTI tractography based on adult macaque and human brains [12]. They showed a substantial improvement with DSI in the mapping of crossing fibers, especially in complex fiber crossing areas such as the optic chiasm, the centrum semi-ovale or the brainstem. However, comparing DTI and more complex approaches is still a poorly understood topic. A better knowledge of the relationship between the diffusion encoding scheme, the fiber orientation estimation method, and the resulting tractography would help in selecting the adequate diffusion scheme for a given application.

In this work, we acquire diffusion MRI data sets with different encoding schemes, and study the influence of the diffusion scheme on the mapping of the human connectome. This presents a challenge, as the methods differ in a number of acquisition parameters such as overall scan time and *b*-value used and as the reconstruction may introduce bias to the results. To be able to compare the results, we need to find a common description. Thus, we aim at comparable SNR properties, apply for all data the same tractography methodology, and use the structural connection matrices as a means to investigate differences between the techniques. It should be noted that the applied diffusion encoding schemes were chosen to match the appropriate models and analysis methods, i.e. DTI, QBI and DSI. In the following, the term *diffusion encoding scheme* will be used to refer to a data set consisting of diffusion images with a specific number of diffusion directions and **q**-space frequencies. This implies subsequently the use of an appropriate model and method to estimate the orientation information.

For the sake of comparability, we aim at reducing the degrees of freedom in reconstruction and tractography, and choose an approach that is publicly available and provides means to deal with all acquired input data. This approach could be criticized as all the aspects acquisition, reconstruction and tractography could be specifically optimized towards a particular goal. In this study, however, we purposely aim at acquiring input data that match in basic conditions (SNR) and we rely on publicly available methodology.

We compare the structural connection matrices obtained from three diffusion schemes. The outcome of the connectivity matrices represents clinically relevant information and – at the same time – this metric allows us to largely omit differences in the methodology that may exist but do not reflect a practical relevance. We evaluate and quantify the performance in terms of brain connectivity by using global network measures and by studying several associative fiber pathways. Using those measurements, we show that the three schemes exhibit clear differences and analyze the factors responsible for those differences. However, we emphasize that ultimately, the choice of the diffusion scheme should be driven by the application.

## Materials and Methods

### 1 MRI Acquisitions

This study was approved by the ethics committee of the Centre Hospitalier Universitaire Vaudois (CHUV, Lausanne, Switzerland). Written informed consent was obtained for all subjects, in accordance with our institutional guidelines. Five healthy female volunteers from 22 to 30 years old were scanned at 3T (Magnetom Trio, a Tim System, Siemens, Germany) using a 32-channel receive head matrix coil. For each subject, eight diffusion acquisitions were performed on three separate days, as follows: four DSI scans with 258 directions, sampling **q**-space by taking the points of a cubic lattice within a hemisphere whose radius is 5 lattice units, three of them with a maximum *b*-value of 8000 s/mm^2^ (DSIq5b8000(1), (2) and (3), acquired on day 1, 2 and 3) and one with 6400 s/mm^2^ (DSIq5b6400 on day 3); one DSI scan with 129 directions, sampling **q**-space by taking the points of a cubic lattice within a hemisphere whose radius is 4 lattice units, with a maximum *b*-value of 6400 s/mm^2^ (DSIq4 on day 2); one HARDI scan with 257 encoding gradients uniformly distributed over a sphere and a maximum *b*-value of 3000 s/mm^2^ (HARDI on day 1, or QBI); two DTI scans with 65 and 21 directions (DTI65 and DTI21 on day 1 and day 3, respectively), with encoding gradients uniformly distributed over a sphere and a maximum *b*-value of 1000 s/mm^2^. The acquisition parameters are summarized in Table 1. Note that the HARDI scan is frequently denoted by the term *QBI* in what follows, in order to give emphasis to the employed reconstruction technique.

**Table 1:** Parameters of the diffusion MR acquisitions.

	DSIq5b8000	DSIq5b6400	DSIq4	HARDI	DTI65	DTI21
TR (ms)	6000					
TE (ms)	138	138	138	110	89	89
Max. *b*-value(s/mm^2^)	8000	6400	6400	3000	1000	1000
Encodinggradients	258	258	129	257	65	21
Acquisitionblock	96×96×34					
Spatialresolution(mm)	2.21×2.21×3					
Number ofaverages	1	1	2	1	4	3
Day	1,2,3	3	2	1	1	3

For all acquisitions, a twice-refocused spin echo sequence [23] with bipolar diffusion encoding gradients and identical imaging parameters was used (repetition time, field of view and spatial resolution). This sequence allows minimization of residual eddy-current effects to a level that has been demonstrated to be negligible for the presented application at the used system [23], [24]. Therefore, post-hoc eddy current correction was not applied. The diffusion encodings in the DSI schemes were implemented in an interleaved fashion, leading to an alternating acquisition of low and high b-value images, which serves an improved qualitative assessment of motion. To maximize the match between the scans with different encoding schemes, the acquisition time was kept constant (approx. 26 minutes), i.e. the DTI65 scan was acquired with four averages leading to 256 (4×64) acquired diffusion directions. Individual repetitions (single averages) as well as the complex averaged images (two and four averages) were used for the comparison analysis. The complex averaging is based on the method presented in [25], however, no phase correction was applied as the scans with the highest diffusion weighting of 8000 s/mm^2^ did not provide enough signal for a reliable phase estimation. Though suboptimal due to the lack of phase correction, it turns out that the used implementation improved the data quality of averaged images. In agreement with recent literature [25] we found that i) compared to magnitude averaging, the complex averaging decreases the noise floor following the square root law and ii) the SNR improvements are very similar to magnitude averaging and only slightly below the expected square root increase as function of number of averages. SNR estimates slightly below the expected square root dependency indicate the presence of residual phase cancellations from bulk head and physiological motion.

**Figure 1:**
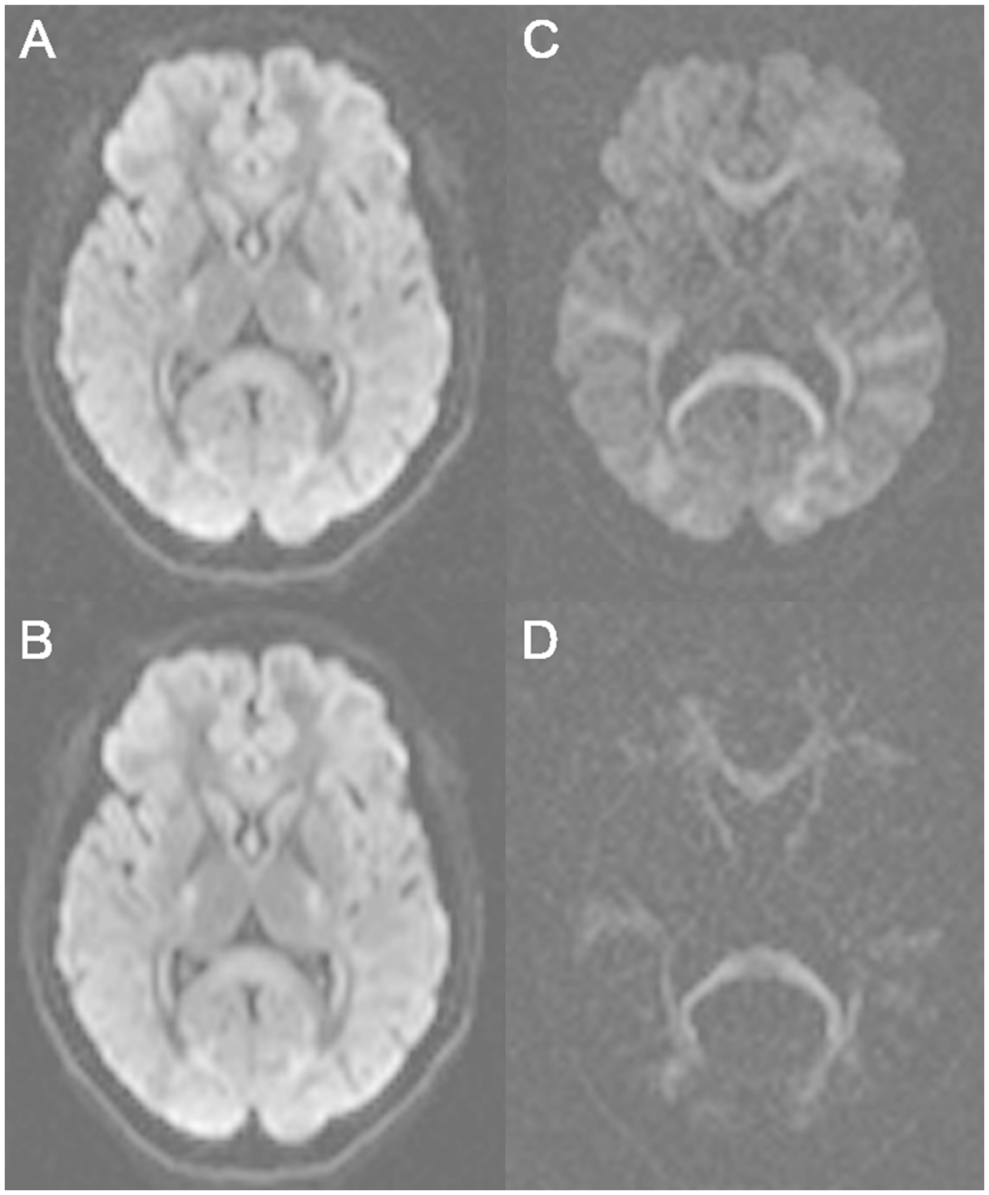
Diffusion-weighted images. Diffusion-weighted images obtained with various *b*-values: b = 1000 s/mm2 single average (A), b = 1000 s/mm2 four averages (B), b = 3000 s/mm2 single average (C), b = 8000 s/mm2 single average (D).

Similarly, the DSIq4 was acquired with two averages leading to 256 (2×128) acquired diffusion directions (in the following we refer to the averages as individual scans). The DTI21 was not SNR-matched and resulted from the averaging of three individual scans, corresponding to an acquisition time of about 6 minutes. Note that all diffusion acquisitions used an identical EPI readout and repetition time. Matching the EPI readout time (SNR) of the individual scans leads to the acquisition of averages for DSIq4, QBI and DTI series. This enables additional analysis steps, i.e. exploring the effects of averaging on the connectome based analysis for those scans. As an illustration, several diffusion-weighted images are depicted in Figure 1.

For anatomical reference, a high-resolution T1-weighted (MP-RAGE) MRI was performed in a matrix of 240×256×160 voxels of 1mm×1mm×1.2mm resolution (TR/TI/TE = 2300/900/2.89ms, iPAT = 2, TA = 5∶12min) [26].

### 2 Structural Connection Matrices

**Figure 2:**
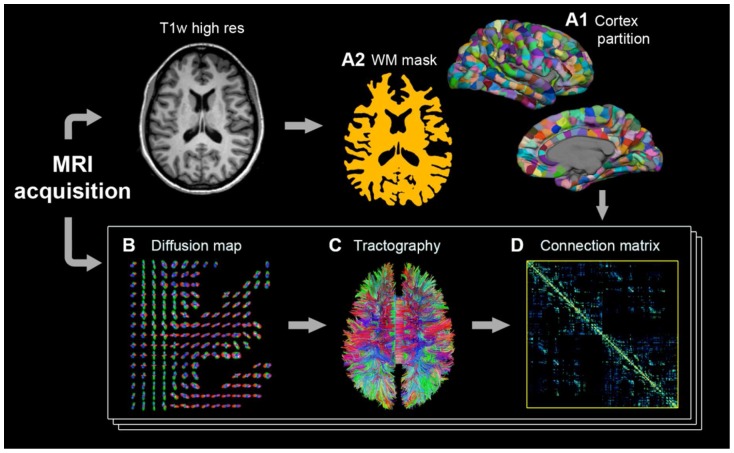
Overview of the methodology. The creation of the structural connection matrices is a four-step process: gray matter partition into regions of interest (ROIs) and white matter mask extraction (A), processing of the diffusion MR acquisitions (B), whole-brain tractography (C) and construction of the connection matrices (D).

The creation of the structural connection matrices follows the four-step process depicted in Figure 2 [27], [28]. First, both the gray and white matter volumes are extracted from the high-resolution T1-weighted acquisition of day 1, and the gray matter (cortical surface and sub-cortical structures) is partitioned into small regions of interest (ROIs). Then, each diffusion MR acquisition (each series) is processed in order to get a diffusion map, i.e. a map containing the orientational information in an adequate form for tractography. The diffusion map is subsequently used to perform whole-brain tractography. Finally, for each subject and for each diffusion MR acquisition, a connection matrix is built by computing the number of fibers connecting every pair of ROIs. Each of these steps is described in what follows.

### 2.1 Extraction and Partition of the Cortical Surface and Sub-cortical Structures

The aim of this step is to partition the cortical surface into ROIs that are compact and of similar size. Ideally, each ROI should be placed in the same anatomical location for each subject, thus allowing inter-subject comparison of the connection matrices. The proposed procedure relies on an atlas-based cortical registration method using the curvature information, i.e. sulcus and gyrus [29], [30]. This method has been directly implemented in the Freesurfer software (http://surfer.nmr.mgh.harvard.edu), providing an automatic labeling of the cortex into 66 gyral-based parcels, which are defined using curvature-based information on 40 manually labeled brains [29].

When studying the human connectome, the partition into those 66 anatomical regions may however not provide a sufficient resolution to evaluate the connectivity locally. In this context, our group recently proposed a subdivision of the original Freesurfer atlas into 998 small ROIs with a surface of about 140mm^2^ [27], [28]. This custom atlas is transferred to the cortical surface of each subject with Freesurfer, by applying the transformation computed for the original atlas-based cortical registration, thus maintaining the topological constraints.

Additionally, the deep gray nuclei (thalamus, pallidum, putamen, caudate nucleus, nucleus accumbens and subthalamus), as well as the hippocampus, the amygdala and the brainstem are identified by an atlas-based segmentation using the same software [31]. Since all these structures are relays for the cerebral fibers, they are considered as a ROIs, leading to a total number of 1015 ROIs.

Furthermore, whole-brain tractography requires a white matter mask which defines the volume in which the virtual fibers are allowed to grow. This white matter mask is obtained by filling the white matter surface and removing the ventricles, the deep gray nuclei, the hippocampus and the amygdala.

The gray matter partition is based on the T1-weighted MP-RAGE acquisition. Since diffusion MR data are acquired using a different field of view and because of the subject’s position variability across scanning sessions, both the cortical partition and the white matter mask need to be transformed to the space of each diffusion MR acquisition. This is achieved using a rigid-body registration method (FLIRT registration tool with 6 degrees of freedom [32], http://www.fmrib.ox.ac.uk/fsl/). Note that this registration does not account for the different distortion properties of the anatomical and the EPI readouts.

### 2.2 Processing of Diffusion MR Acquisitions

Before reconstruction of the orientation information, image quality of each diffusion series was manually inspected visually for subject motion. The first processing of the raw images from each diffusion series comprised the registration to the anatomical MPRAGE scan using an affine registration to remove inter-series subject motion.

Each diffusion MR acquisition has to be processed to produce a diffusion map containing the orientational information, such that it is suitable for whole-brain tractography. The reconstruction technique is specific to the scheme used for the diffusion MR acquisition. For DSI data, reconstruction of the data is achieved according to the DSI protocol [16] as follows: the diffusion spectrum is obtained by taking the Fourier transform of the **q**-space MR signal. Next, the diffusion spectrum is radially projected, yielding an Orientation Density Function (ODF). In other words, the ODF extracts the angular structure of the diffusion spectrum. In the case of HARDI, data are processed according to the QBI technique, i.e. using the Funk-Radon transform, which was shown to provide a good approximation to the ODF [20]. Finally, DTI data are reconstructed by fitting a second-order symmetric tensor, describing the diffusion along each direction [11]. All these reconstruction techniques are implemented in the Diffusion Toolkit [33] (http://www.trackvis.org/dtk), allowing an automatic processing of diffusion MR data. The diffusion toolkit is used for all reconstructions as it parameterized all reconstructions in a way that they provide ODFs and/or tensor fields optimized for streamline tractography. In the context of the goals of this study, this provides a fair base for comparison performed across the various diffusion scans.

### 2.3 Whole-brain Tractography

Whole-brain tractography is performed using a streamline algorithm which creates virtual fibers in the brain white matter, estimating the trajectories of real axonal bundles [8], [12], [34], as described below.

First, in each white matter voxel a set of normalized direction vectors is extracted, corresponding to the local maxima of diffusion. For DSI and QBI data, this is achieved by identifying the local maxima of the ODF. In the case of DTI a unique vector is obtained corresponding to the first eigenvector of the tensor. Then, in each voxel a set of uniformly distributed initialization points is chosen according to a random process. The number of points is arbitrarily set to 32*n_v_*, with *n_v_* the number of direction vectors in voxel *v*. Next, from each initialization point a fiber growth process is started in two opposite directions using a fixed step size of 1mm, locally following the direction vector that is the closest to the current fiber trajectory. To avoid abrupt changes of direction, the process is aborted if it results in a change of trajectory sharper than 60 degrees/mm. The growth process is stopped when both end-points of the virtual fiber have left the white matter.

Additional fiber post-filtering is performed as follows. A length threshold is applied, such that all fibers shorter than 5mm are eliminated. Very short fibers (range <5 mm) are indeed not considered as relevant information as we mainly focus on the major fiber pathways. The same procedure is applied to fibers longer than 200mm, which are unlikely to represent realistic axonal pathways considering the field of view of the MR acquisitions. Finally, a fiber is kept only if both end-points lie in one of the ROIs obtained with the previous step. After filtering, each tractography experiment results in approximately 0.4 to 0.6 million virtual fibers. Note that this quantity results from an arbitrary setting of the number of initialization points for tractography.

### 2.4 Construction of the Connection Matrices

Combining the gray matter partition and the whole-brain tractography described previously [27], [28], we can identify the set of fibers *F(i, j)* connecting each pair of ROIs *i* and *j*. We then collect this information in a connection matrix, where each cell *M(i, j)* contains the number of fibers connecting the ROIs *i* and *j*. Note that the diagonal of the connection matrix is arbitrarily set to zero, i.e. all fibers that link a given ROI to itself are discarded. Note that the connectivity as it is measured in this work is similar to the definition of the connectivity proposed by Yo et al. for deterministic tractography approaches [35]. However, since our ROIs all have similar sizes, we do not normalize the connectivity by the surface of the ROIs.

In what follows, we use the term *fiber* when referring to a single trajectory produced by tractography. In contrast, the set of fibers connecting a given pair of ROIs is denoted by the term *connection*. Additionally, we also introduce the matrix of the connection distance *d(i, j)*, defined as the geodesic distance in the brain white matter (i.e., the shortest path being confined in the white matter volume) separating the ROIs *i* and *j*. This metric turns out to be essential to the analysis of the connection matrices.

Since we aim at studying the influence of the diffusion encoding scheme on the connection matrices obtained with tractography, it is essential to 1) maximize the comparability across the diffusion schemes and 2) minimize the differences in the methodology. First, we use identical EPI readout duration and repetition time for all diffusion acquisitions and we use complex averaging to keep the scan time constant. For the reconstruction of the pixel-wise tensor or ODF the respective SNR is scaling with the square root of the number and duration of the EPI readout. Thus, a match of scan time represents an attempt to equalize the different diffusion acquisitions. Second, the processing of the diffusion data is based on a software package that is suited for all the diffusion encoding schemes used in this study. This ensures that the produced diffusion maps have identical properties. The other steps of the methodology are independent from the diffusion acquisitions and applied equally (i.e. with the same parameters) to all diffusion maps (e.g. registration of the gray matter partition and tractography).

## Results

### 1 Visual Inspection of All Scans

Qualitatively all scans are rated of no or very low motion parameters. As mentioned previously, the diffusion encodings in the DSI schemes are implemented in an interleaved fashion, leading to an alternating acquisition of low and high b-value images. Thus, visual inspection of an entire DSI scan resorted by ascending b-value allows reasonable qualitative assessment of motion throughout the entire experiment.

### 2 The Relationship Between Connectivity and Diffusion Encoding Scheme

**Figure 3:**
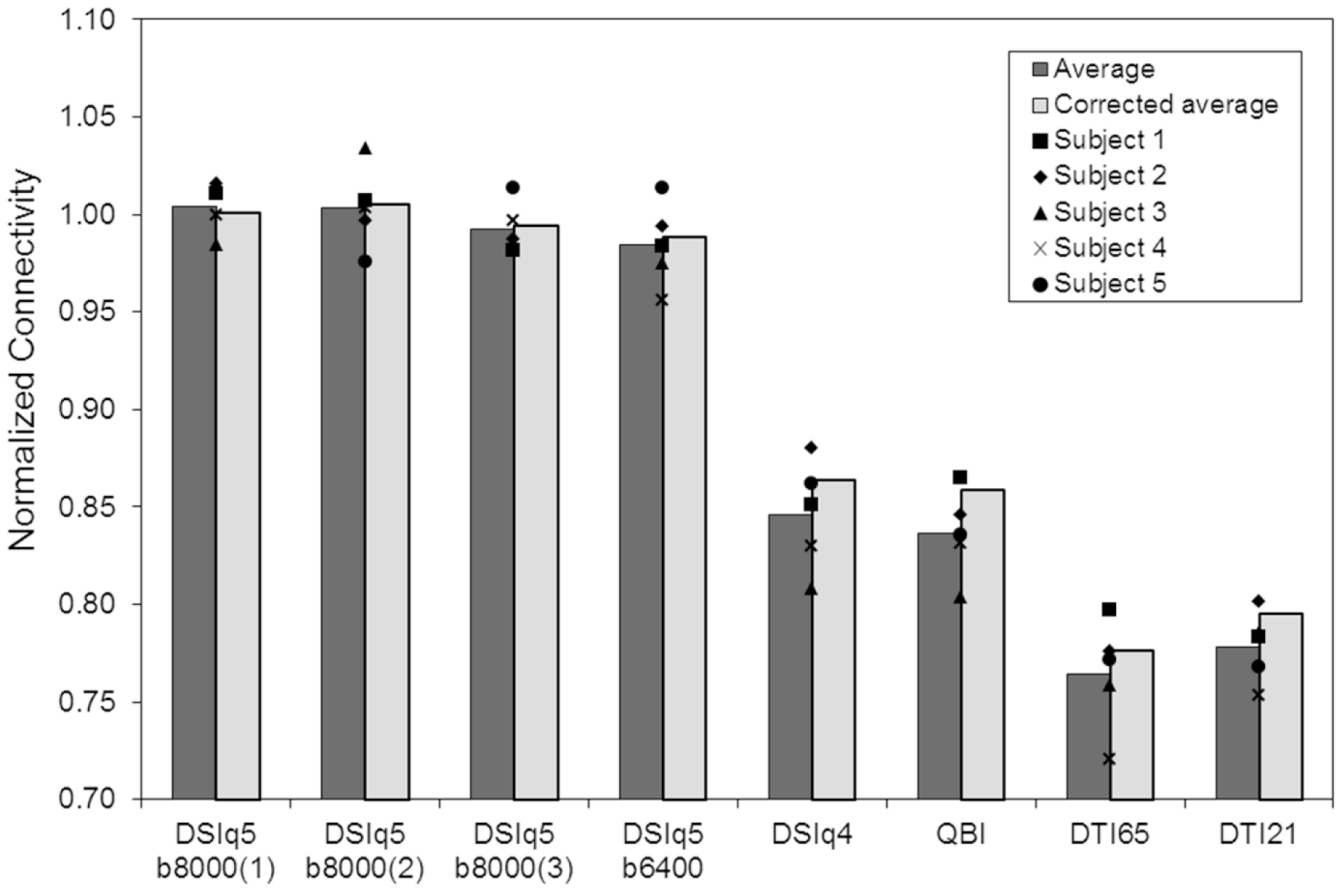
Normalized connectivity as a function of the diffusion encoding scheme. Dark gray bars represent averages across the five subjects and symbols indicate data for individual subjects. Light gray bars represent the averaged normalized connectivity after fiber limitation, ensuring that every connection matrix of an individual subject is built using the same number of fibers.

We evaluate the connectivity produced by each diffusion encoding scheme using the number of computed connections, i.e. the number of links between a pair of ROIs given by at least one fiber. The results of the whole brain analysis for the individual subjects are reported in Table 2. To account for physiological and anatomical differences across individuals, we define the normalized connectivity as the ratio between the number of connections computed from any scan and the average connections of the three DSIq5b8000 scans. The relationship between the normalized connectivity and the diffusion encoding scheme is depicted in Figure 3. Additionally, we perform paired t-tests on the number of connections under the null hypothesis that the samples come from distribution with equal means, and report the corresponding p-values in Table 3.

**Table 2:** Number of connections for the individual subjects.

	DSIq5b8000(1)	DSIq5b8000(2)	DSIq5b8000(3)	DSIq5b6400	DSIq4	QBI	DTI65	DTI21
Subject 1	12755	12709	12386	12411	10736	10915	10052	9882
Subject 2	14119	13848	13720	13812	12227	11759	10778	11134
Subject 3	13570	14256	13535	13441	11142	11084	10456	10829
Subject 4	13543	13597	13504	12952	11238	11258	9760	10206
Subject 5	13757	13282	13800	13795	11737	11372	10506	10455

**Table 3:** P-values obtained for paired t-tests performed on the number of connections.

	DSIq5b8000(1)	DSIq5b8000(2)	DSIq5b8000(3)	DSIq5b6400	DSIq4	QBI	DTI65	DTI21
DSIq5b8000(1)	–	0.96*	0.16*	0.07*	2.9E-05	3.6E-05	5.0E-05	1.3E-05
DSIq5b8000(2)	0.96*	–	0.50*	0.34*	1.8E-03	7.7E-04	2.1E-04	3.8E-05
DSIq5b8000(3)	0.16*	0.50*	–	0.41*	3.4E-04	3.2E-04	1.8E-04	8.8E-05
DSIq5b6400	0.07*	0.34*	0.41*	–	1.6E-04	3.8E-04	5.3E-05	4.3E-05
DSIq4	2.9E-05	1.8E-03	3.4E-04	1.6E-04	–	0.32*	3.3E-03	5.2E-03
QBI	3.6E-05	7.7E-04	3.2E-04	3.8E-04	0.32*	–	2.6E-03	6.8E-03
DTI65	5.0E-05	2.1E-04	1.8E-04	5.3E-05	3.3E-03	2.6E-03	–	0.20*
DTI21	1.3E-05	3.8E-05	8.8E-05	4.3E-05	5.2E-03	6.8E-03	0.20*	–

Null hypothesis: the samples come from distributions with equal means. The * symbol indicates the pairs of diffusion encoding schemes for which the null hypothesis cannot be rejected (p>0.05).

The normalized connectivity is found to be very similar across the three DSIq5b8000 scans, with a variation of only 5.2%. The normalized connectivity obtained with the DSIq5b6400 scan is 98.4% (averaged over the five subjects). Accordingly, the null hypothesis cannot be rejected for the number of connections between the four DSIq5 scans at p = 0.05. The averaged intra-subject scan-rescan variability, evaluated by computing the standard deviation across the three DSIq5b8000 scans (σ = 229), is found to be smaller than the averaged inter-subject variability (σ = 533). Note that the scan-rescan reproducibility is performed for DSIq5b8000 only, as the entire scanning protocol was being performed on three separate days due to the extensive scan time. The scan-rescan reproducibility of the DSIq5 scan is used to ensure the comparability of the data rather than as a comparison metric across the various diffusion encoding schemes.

The DSIq4, QBI and DTI scans result in a significantly lower normalized connectivity than the DSIq5 scans (p≤1.8e-3). Those results are unchanged after a Bonferroni correction, except for the pair DSIq4– DSIq5b8000(2) for which the null hypothesis cannot be rejected. We note that DSIq4 and QBI scans produce similar results, with a normalized connectivity of 84.6% and 83.6% (averaged over the five subjects), respectively. The paired t-tests reveal that the differences between those two diffusion schemes are not significant (p = 0.32). The DTI64 and DTI20 scans produce the lowest normalized connectivity overall, with an average of 76.5% and 77.8%, respectively. In this case again, the null hypothesis cannot be rejected (p = 0.20), but the two scan variants differ significantly from both the DSIq5 (p≤2.1e-4) and the DSIq4/QBI (p≤6.8e-3) schemes. We note however that after a Bonferroni correction, the differences between DTI and DSIq4/QBI scans do not remain significant. Additional results obtained with the DTI scans are presented in the next section.

In the results presented above, a connection is considered if there is at least one virtual fiber between the corresponding pair of ROIs. The added connections observed for the DSIq5 scans might thus be due to spurious fibers (i.e. single tracts generated artifactually), and hence not representing meaningful connections. To rule out this possibility, we repeat our experiments by considering only the connections consisting of at least *n_f_* fibers, with *n_f_* = {5, 20}. Our observations remain valid for these two connectivity thresholds, suggesting that the added connections observed for the DSIq5 scans are not spurious connections (see Tables S1–S4).

With the proposed tractography algorithm, the number of generated fiber tracts is proportional to the average number of diffusion directions per voxel, which depends on the type of diffusion encoding scheme. The higher connectivity (defined as higher number of connections) observed for DSI and QBI scans could thus result artifactually from the higher number of seed points per voxel. To rule out this possibility, we apply a fiber limitation as follows. First, we choose a fixed value *F_s_* defined as the minimum number of fibers produced for subject *s* across all diffusion schemes. Then, for each subject and diffusion scheme, a subset of *F_s_* fibers is randomly chosen prior to the computation of the connection matrix. This ensures that every connection matrix of an individual subject is built using the same number of fibers. Although a small increase of the averaged normalized connectivity can be observed with the fiber limitation for the DSIq4, QBI and DTI scans (up to 2.2%), the reported results are essentially unchanged (Figure 3, light gray bars). Consequently, the reported connectivity differences cannot be attributed to the number of generated fiber tracts, implying that the connectivity results are largely decoupled from the number of seed points per voxel.

### 3 The Effect of Complex Averaging

As previously mentioned, we use complex averaging as a way to maximize the SNR [25]. Averaged DSIq4 and DTI65 data are thus computed using 2 and 4 individual scans, respectively, but also the individual scans that form the SNR matched DSIq4 and QBI experiment are investigated. Note the four times reduced scan time for the individual scans (6.5 vs. 26 minutes). DTI21 data result from an averaging of three individual scans only, and consequently have comparable SNR properties to the individual scans of DTI65. In what follows, we analyze the influence of the averaging on the connectivity, by comparing the results from individual scans with those obtained after averaging.

We analyze the normalized connectivity obtained from the following data sets: DSIq4 individual scans and averaging of two scans, DTI21 individual scans and averaging of two and three scans, DTI65 individual scans and averaging of two, three and four scans. For DTI data, the normalized connectivity is found to be very similar, not only for the various levels of averaging, but also across DTI21 and DTI65 data sets. This is confirmed by paired t-tests on the number of connections, showing that the DTI scans cannot be statistically differentiated from each other (p≥0.19 for all pairs of DTI data sets). However, we observe a trend of higher variance when averaging scans and when going to higher directional encodings. The normalized connectivity obtained with the DSIq4 scheme is found to be higher for the individual scans than for the averaged acquisitions (2.4% on average). Although the observed difference is small it remains statistically significant, as confirmed by the paired-t test on the number of connections (p = 3.85e-4).

### 4 The Role of Connection Distance

**Figure 4:**
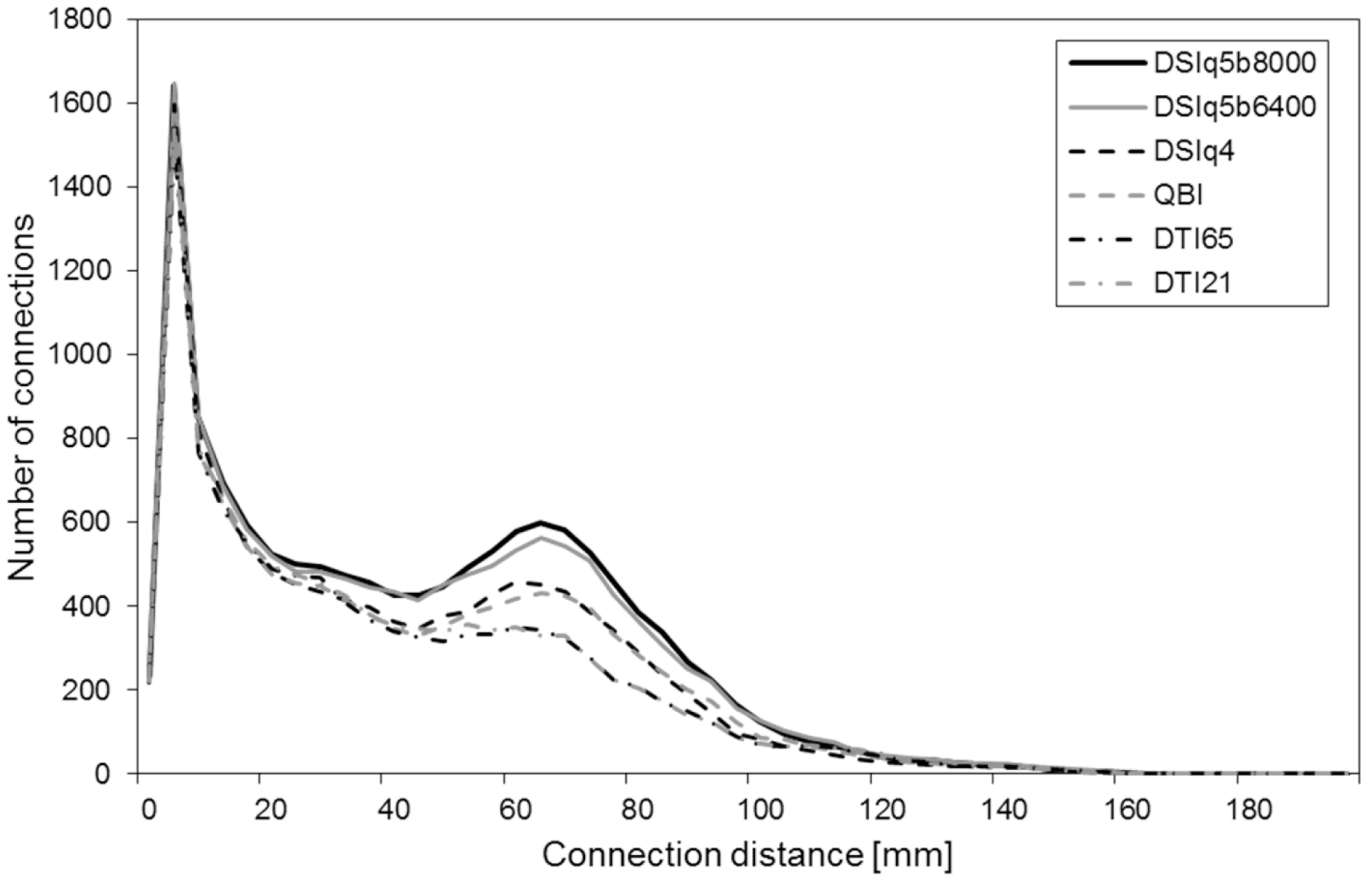
Number of connections as a function of the connection distance. The connection distance *d(i, j)* is defined as the geodesic distance in the brain white matter separating the regions of interest (ROIs) *i* and *j*. These results are obtained by averaging over the five subjects.

Next, we focus on the connection distance, i.e. the distance along a fiber path between a pair of ROIs. The connection distance has been previously defined as the geodesic distance in the white matter separating each pair of ROIs [36]. For each connection matrix, we collect the set of distances associated with the obtained connections. We report in Figure 4 the connection distance distribution for the various diffusion encoding schemes (averaged over the five subjects). Although the distributions reveal similar characteristics, the DSIq5 scans show a heavier tail compared to the DTI scans (see distances >50mm). The DSIq4 and QBI scans lie between the DTI and DSIq5.

Moreover, we notice that the differences across diffusion schemes are mainly found in the range 50–100mm. To confirm this observation, we compute the averaged normalized connectivity in the following categories of connection distance: 0–50mm, 50–100mm and 100–200mm. The results obtained for each diffusion scheme are reported in Table 4. The normalized connectivity in the range 0–50mm is above 89% for all diffusion schemes. For the range 50–100mm it is only approximately 76% for DSIq4 and QBI, and 59% for DTI65 and DTI21. In the range 100–200mm the normalized connectivity is above 81% for QBI and DTI scans, but only of 66% for the DSIq4 scan. However, due to the small proportion of long connections in the data sets the variability is particularly high in the range 100–200mm. The corresponding results should thus be interpreted with caution.

**Table 4:** Normalized connectivity as a function of the diffusion encoding scheme.

	DSIq5 b8000(1)	DSIq5 b8000(2)	DSIq5 b8000(3)	DSIq5 b6400	DSIq4	QBI	DTI65	DTI21
0–50mm	1.00	1.00	1.00	0.99	0.92	0.89	0.89	0.91
50–100mm	1.02	1.00	0.98	0.96	0.77	0.76	0.59	0.59
100–200mm	0.97	1.06	0.97	1.06	0.66	0.81	0.81	0.83

Three categories of connection distances: 0–50mm, 50–100mm and 100–200mm. The reported values are obtained by averaging the normalized connectivity over the five subjects.

### 5 The Impact on Selected Associative Fiber Pathways

**Figure 5:**
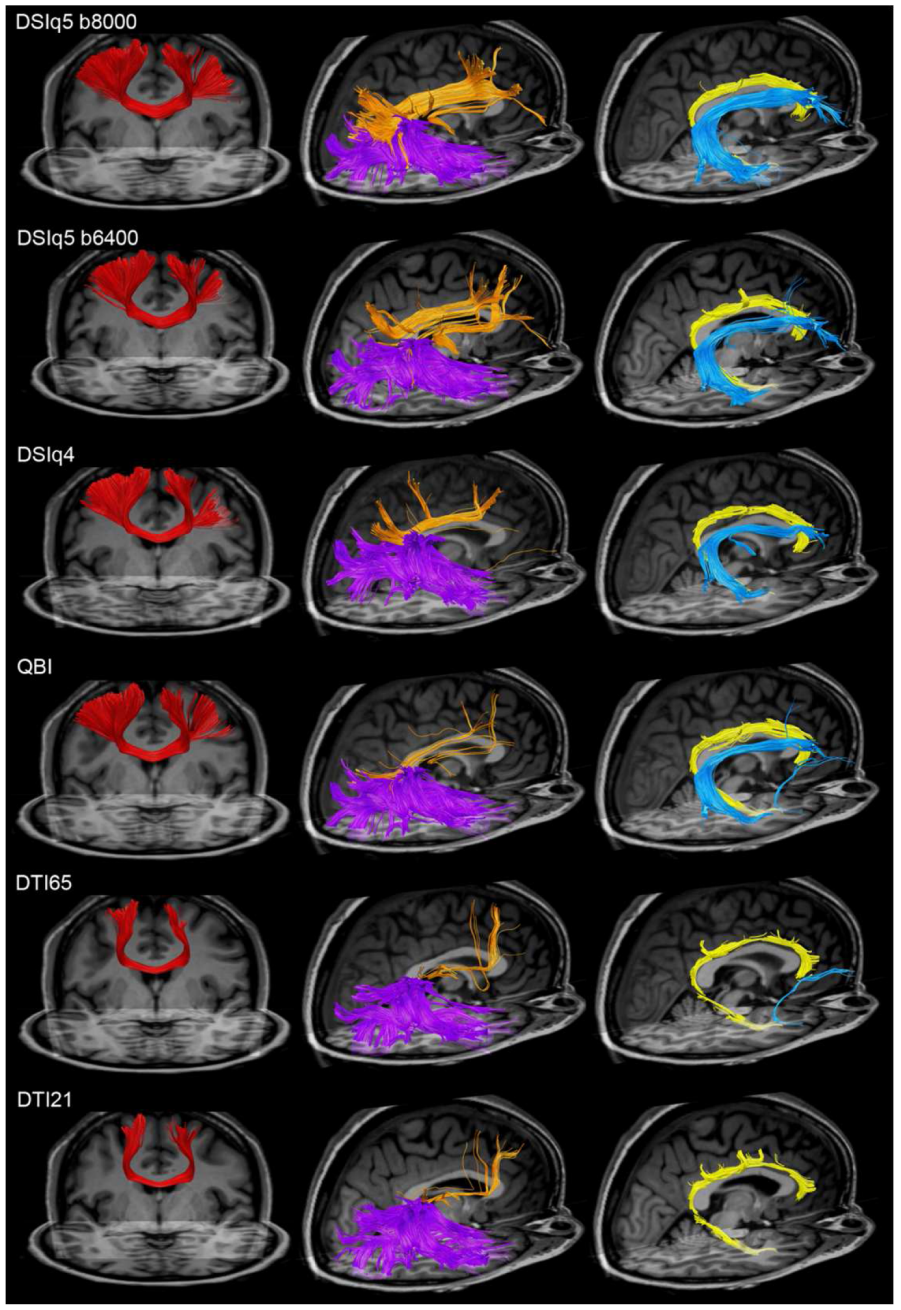
Mapping of some associative fiber pathways. Mapping of the primary motor cortex projections (red), inferior longitudinal fasciculus (violet), superior longitudinal fasciculus (orange), arcuate fasciculus (blue) and cingulum bundle (yellow), for the various diffusion encoding schemes (subject 1).

**Figure 6:**
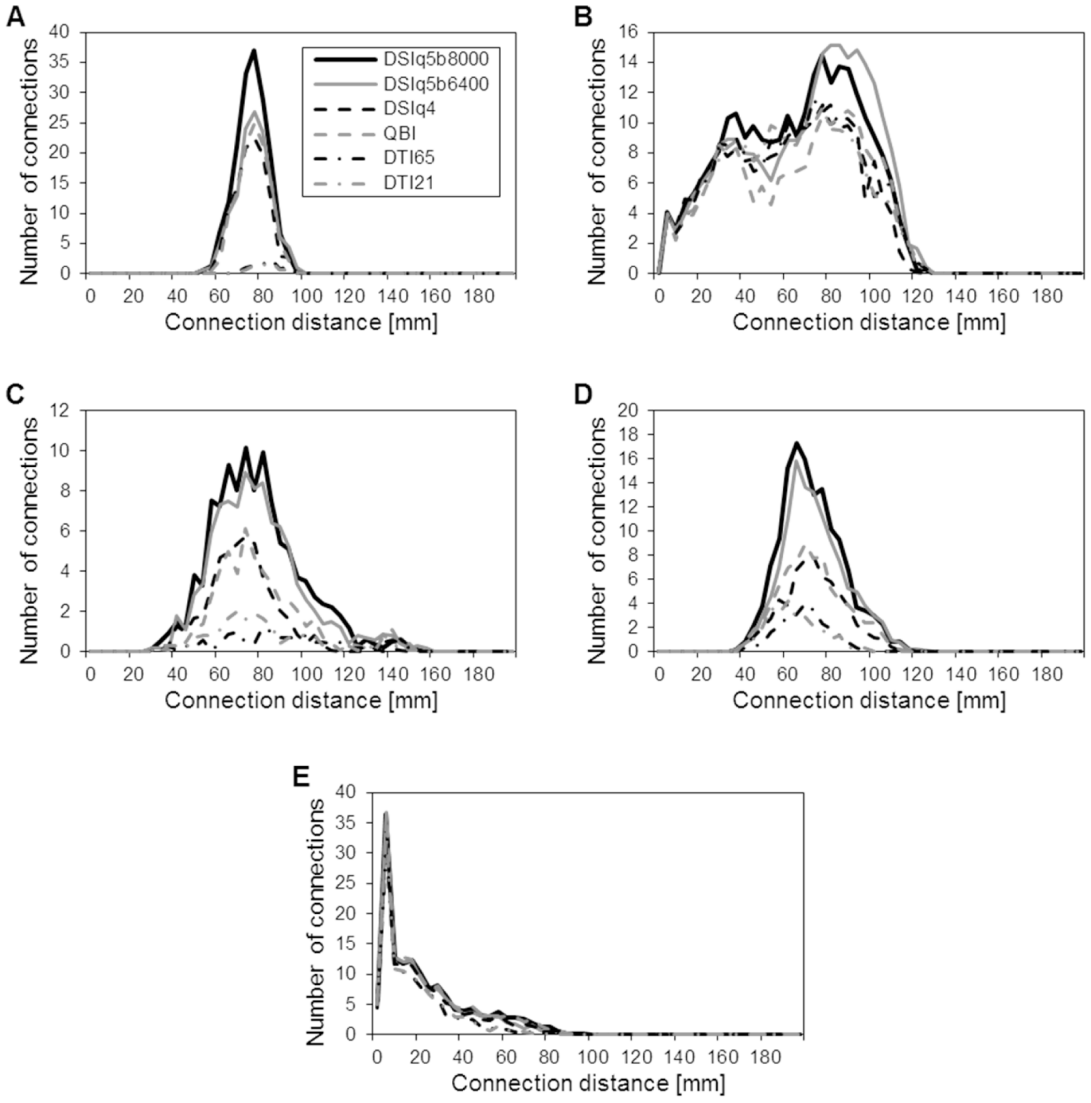
Connection distance distributions for the selected associative fiber pathways. Distribution of connection distances for the primary motor cortex projections (A), inferior longitudinal fasciculus (B), superior longitudinal fasciculus (C), arcuate fasciculus (D) and cingulum (E). These results are averaged over the five subjects.

As an illustration, we select and identify several major association fiber pathways, as follows. Primary motor cortex (PMC) projections connect the left and right precentral gyri through the corpus callosum. The inferior longitudinal fasciculus consists of the fibers originating from the temporal lobe and ending in the occipitoparietal junction or in the occipital lobe. The superior longitudinal fasciculus connects the inferior and superior parietal lobules with the middle and superior frontal gyri. The arcuate fasciculus projects from pars triangularis, pars opercularis and the rostral part of the middle frontal gyrus to the superior, middle and inferior temporal gyri. Finally, the cingulum bundle comprises the fibers interrelating the cingulate and parahippocampal gyri. All those regions are identified using the gyral-based atlas from Freesurfer. In Figure 5, we plot the above-mentioned fiber pathways for the various diffusion encoding schemes of a single subject. Additionally, we report in Table 5 the averaged normalized connectivity for the fiber pathways of primary motor cortex projections, inferior longitudinal fasciculus, superior longitudinal fasciculus, arcuate fasciculus and cingulum bundle (for reference see Figure 5). In Figure 6, we show the corresponding distributions of connection distances for the five fiber pathways.

**Table 5:** Normalized connectivity as a function of the diffusion encoding scheme for the selected association fiber pathways.

	DSIq5 b8000(1)	DSIq5 b8000(2)	DSIq5 b8000(3)	DSIq5 b6400	DSIq4	QBI	DTI65	DTI21
PMC proj.	1.02	1.01	0.97	0.81	0.71	0.73	0.05	0.04
Inf. long. fasc.	1.00	0.96	1.04	1.03	0.81	0.77	0.83	0.80
Sup. long. fasc.	1.08	0.98	0.94	0.92	0.48	0.51	0.14	0.25
Arcuate fasc.	1.00	0.99	1.01	0.87	0.47	0.54	0.23	0.28
Cingulum	1.04	0.99	0.97	0.99	0.95	1.04	0.75	0.76

The reported values are obtained by averaging the normalized connectivity over the five subjects.

With DSI and QBI scans, the primary motor cortex projections are widely distributed throughout the precentral gyri, from the apex down to its lower limit at the lateral sulcus. In contrast, only the apical part of the precentral gyri is connected with the DTI data sets. This is confirmed by a strongly decreased normalized connectivity for the DTI scans (below 5%). The superior longitudinal and arcuate fasciculi show a lower connectivity for DSIq4 and QBI (around 50%) than with DSIq5. This is explained by the fact that the fibers capture the global shape of the tract but are less spread throughout the origin and destination regions. The normalized connectivity obtained for these fiber tracts is further decreased with the DTI scans (between 14% and 28%), indicating that the corresponding fiber pathways are difficult to identify with this type of diffusion scheme. For example, we see in Figure 5 that the right arcuate fasciculus cannot be retrieved with the DTI scans of subject one, and that the superior longitudinal fasciculus is only partially mapped. It is important to note that these three fiber pathways mainly consist of connections whose distance lies in the range 50–100mm, as confirmed in Figure 6.

In contrast, the inferior longitudinal fasciculus and the cingulum are mapped consistently across scans (Figure 5), with an averaged normalized connectivity above 75% for all diffusion encoding schemes, as shown in Table 5. Looking at the corresponding distributions of connection distances in Figure 6, we notice that the inferior longitudinal fasciculus mainly consists of connections lying in the range 20–100mm, and most of the connections that form the cingulum have a distance shorter than 50mm.

## Discussion

Earlier work has shown that high angular resolution diffusion techniques improve the mapping of fiber pathways in complex crossing areas as compared to DTI [12]. It has been also suggested that QBI and DSI schemes increase the sensitivity of tractography [13]. Recently, Yo et al. have compared several reconstruction techniques (DTI, CSD, ball-and-stick, persistent angular structure) by using connection matrices resulting from tractography on a selected set of regions of the human brain [35]. They have shown that fiber crossing models reveal more connections than the simple tensor model. The present study aims at gaining a better understanding of the influence of the diffusion encoding scheme on the performance of whole-brain tractography.

Our first finding is that DSIq5 scans, acquiring 258 encoding directions in the **q**-space, maximize the number of connections in the connection matrices when comparing with other diffusion schemes employing fewer encoding directions at lower angular resolution. These findings have to be interpreted with care as we have no means to assess the underlying specificity of the connections. The observation of more fibers cannot be interpreted as a better description of the underlying brain structure. However, correlations between brain connectivity measures using DSI and fMRI suggest a high specificity of the fibers detected with DSI [37]. Moreover, the analysis of several well-known associative fiber pathways strongly suggests that the added connections correspond to real anatomical fiber tracts and lead to improved tracking accuracy, rather than solely adding noise. These results suggest that the DSI technique may provide a higher sensitivity to map the fiber pathways in brain white matter. The level of sensitivity improvements, however, depends on the fiber pathways under investigation. As the higher diffusion encoding directions provide the strongest advantage for pathways in the 50–100mm range, differences between the diffusion encoding schemes naturally will taper off in pathways that extend beyond this range.

On the other hand, we show that the performances of the DTI scans are strongly limited by the underlying Gaussian model: about one fourth of the connections obtained with the DSIq5 scan are not mapped with DTI, even as high as 40% in the distance range 50–100mm. The consequences are important, since even well-known association bundles, such as the arcuate fasciculus or the superior longitudinal fasciculus, are not as comprehensively mapped. The additional diffusion schemes, namely the DSIq4 and QBI, exhibit intermediate results.

Interestingly, the QBI and DSIq4 scans perform similarly although the diffusion schemes are fundamentally different: the HARDI scheme used for QBI is based on the acquisition of the diffusion signal on a single shell in the **q**-space at a moderate *b*-value (typically 3000mm/s^2^) [38], whereas the DSIq4 scheme acquires the signal on multiple shells with a high maximum *b*-value. In theory, the higher *b*-value used for DSIq4 scans may provide a higher angular resolution as compared to HARDI acquisitions, at the cost of an increased amount of noise. Our results tend to indicate that the potential gain associated to the additive orientational information is counteracted by the higher level of noise and potentially motion, i.e. with longer acquisition durations. Moreover, we notice that the addition of a fifth shell in the **q**-space (yielding a DSIq5 scan) substantially increases the resulting connectivity. Small to moderate changes of the maximum *b*-value seem, however, to have only a limited impact on the connection matrices, as supported by the comparison of the DSIq5b8000 and DSIq5b6400 scans.

Our second finding is that the biggest differences between the diffusion encoding schemes are found for mid-range connections, i.e. connections with a length between 50 and 100 mm. Those connections consist mainly in 1) neighboring association fibers connecting pair of regions inside the same lobe, and 2) long-range associative, striatal and commissural fiber tracts [39], [40]. Neighboring association fibers are precisely non-dominant fiber populations, which high angular resolution is theoretically capable of disentangling. The long-range association fibers are composed of relatively big and tightly packed axonal bundles, and are consequently dominant compared to the smaller fiber tracts that may cross their trajectory. However, we know from anatomy that many long-range connections also cross each other in large fiber crossing areas, such as the centrum semi-ovale. This is the case of the primary motor cortex projections, the arcuate fasciculus and the superior longitudinal fasciculus. Our results show that those tracts are only partially mapped with the DTI scans. This suggests that HARDI and DSI acquisitions are not only required to map non-dominant fiber populations, they also improve the mapping of the major associative fiber pathways.

In contrast, the connectivity in the range 0–50mm is found to be similar for all the diffusion schemes. These connections mainly consist in short association fibers, connecting pairs of regions inside the same or adjacent gyrus. In a previous study, we showed that short-range connectivity (typically in the range 0–40mm) is partially due to random effects, which arise in any tractography experiment independent of the diffusion scheme [36]. Due to the lack of anatomical knowledge about the short associative fiber pathways, we are not able to infer whether the produced fibers reflect the true underlying connectivity or noise. Consequently, it is not possible for this category of connections to evaluate the differences that may exist between the various diffusion encoding schemes.

Our third finding is that the complex averaging of diffusion acquisitions, as applied for the DSIq4 and DTI scans, does not improve the resulting tractography. The connectivity is even significantly decreased for the averaged DSIq4 scans as compared to the single scans (p = 3.85e-4), although the signal-to-noise ratio is better. The negative impact of the averaging in the case of the DSIq4 scans may be explained by a presumably mild bulk subject motion that is not taken care of due to the low signal in the diffusion weighted images at the highest b-values despite the fact that the background noise is found to be successfully reduced. More recent technology developments will allow to overcome those limits in future studies [41].

Indeed, it should be noted that the acquisition duration was 25.5 minutes. Thus even mild bulk subject motion and also physiologic noise arising from cardiac and respiration cycle, that will lead to residual phase cancellations, may affect the scans, where the same q-space point is sampled repetitively. When applying complex averaging, these effects will lead to phase cancellation artifacts that could be responsible for the observation of decreases in connectivity measures when averaging data.

For DTI scans, our results suggest that the number of encoding gradients (20 for a single DTI21 data set) already acts as a sufficient averaging factor when reconstructing the tensors. Jones, and Papadakis et al., had shown that the minimum number of unique sampling orientations required for a robust estimate of anisotropy and mean diffusivity was 20 and 30, respectively [42], [43]. Jones however recommended the use of 30 directions or more to estimate the principal direction of diffusion. The results we obtained tend to show that although we might have a higher uncertainty on the tensor orientation with DTI21 scans, the impact at the level of whole-brain tractography is limited: the number of connections produced is similar between DTI21 and DTI65, and the analysis of the major associative fiber pathways did not reveal any differences. The added encoding gradients available with a DTI65 scan would thus provide no benefit at the level of whole-brain tractography. Further work would however be required to understand this phenomenon in detail.

Nevertheless, this observation is of high significance in our study. First, it shows that if a long acquisition time is available, the acquisition of an additional shell in the **q**-space (i.e. a DSIq5 scan) is preferable to an averaging of several DSIq4 scans. Second, a DSIq4 experiment requires a scan time of 12–13 minutes and a single-average DTI21 scan can be performed in 2 minutes. This is clinically more realistic than a full DSIq5 of more than 25 minutes acquisition time. Consequently, we want to emphasize that the choice of the optimal diffusion scheme strongly depends on the application.

For clinical applications for which the acquisition time is an issue and which aim at creating scalar maps such as fractional anisotropy maps, a DTI scan can be the most adequate solution. Similarly, to answer some basic questions using tractography results, DTI can produce credible-appearing tracks. However, it is necessary to be extremely cautious when considering fiber tracts running through large fiber crossing areas, such as the arcuate fasciculus or the superior longitudinal fasciculus. In that case, a DSIq4 scan with an acquisition time of approximately 12 minutes should provide enough contrast to accurately identify the major bundles of the brain, and may turn out to be an advisable compromise between angular resolution and acquisition time.

As shown in this work, particular caution with respect to the choice of the diffusion scheme has to be used when investigating tracts of mid-range distance, which partly consist in non-dominant fiber populations. For example, the neighboring association fibers are of high interest in the study of plasticity, in the case of specific networks involving areas nearby, e.g. motor circuits in patients after stroke [44]. In this context, HARDI-based approaches and DSI may be promising techniques to investigate the modification of the connectivity. For such studies, our observations show that the use of a DSI scan with 258 or more encoding gradients is preferable, though this needs more careful control of data quality. Due to the particularly long scan time of this technique (25 minutes and more), it becomes very prone to motion artifacts that may degrade the accuracy and the sensitivity of the method.

### 1 Methodological Considerations

A huge variability of protocol settings including the number of encoding gradients or the maximal *b*-value is reported in the literature: for DTI, a *b*-value between 700 and 1300s/mm^2^ and a number of encoding gradients ranging from 32 to 64 are commonly used and reported [45]–[47]. For QBI, a *b*-value between 2500 and 3000s/mm^2^ is recommended [38], with approximately 250 directions [22], [48]. Typical DSI scans are achieved with 258 (DSIq5) encoding gradients with a maximum b-value ranging between 8000 and 9000s/mm^2^ [28], [49], [50]. Although changes in the protocols may slightly affect the results, it is highly likely that our observations remain valid for the range of typical settings, as suggested by the similar results obtained for the DSIq5b8000 and DSIq5b6400 scans.

Due to the long acquisition time used in this study, the subjects are scanned on three separate days, which lets us assess also certain aspects of the scan-rescan reproducibility. To this purpose, a DSIq5 scan is performed during each scanning session. We remember that the proposed methodology processes each scan independently. The measures obtained on the three DSIq5 scans thus allow us to measure the variability across scans. The results show that 1) the normalized connectivity only varies by 2% on average across the scanning sessions and 2) the scan-rescan variability is smaller than the inter-subject variability (σ = 229 vs. σ = 533). This demonstrates excellent scan-rescan reproducibility and indicates that we have high quality data at each time point. Consequently, none of the reported results can be accounted for by the variability across scanning sessions.

The approach that we propose for the partition of the cortical surface deserves comment. As previously mentioned, this partition is based on an atlas-based cortical registration method that has already been extensively validated [29]. The original atlas is then further subdivided into many small ROIs. The Freesurfer framework maintains the topological constraints when applying the cortical registration, ensuring that the small ROIs are located in the same gyral-based region of the original Freesurfer atlas for every subject. It is nevertheless not guaranteed that each small ROI exactly corresponds to the same anatomical location in every subject, as recently shown by our group [27]. Several factors are responsible for this variability, such as natural inter-subject variations and methodological limitations. However, the present study exclusively relies on network measures which average the connectivity information globally. In this context the proposed approach is not a limiting factor, as suggested by the powerful analyses already performed with a similar methodology [28], [37].

The choice of the tractography algorithm is also crucial, since several constraints have to be considered. First, as we wanted the tractography algorithm to be the same for all diffusion scans, we chose an algorithm that is suited for all diffusion schemes and that does not favor a specific type of input data. Second, we need an algorithm whose parameterization is simple, i.e. 1) it does not require adaptation depending on the type of diffusion schemes, and 2) it has no major influence on the sensitivity and specificity of the tractography. The chosen streamline algorithm meets all those requirements while being computationally very simple. Thirdly, we used a methodology that is freely available to the research community.

It is likely that more advanced tractography algorithms will emphasize differences between the diffusion encoding schemes. At the same time, ongoing research indicates that more efficient diffusion encoding schemes could be employed [21], [51]–[53], that may themselves influence results in addition to any effects of reduced motion sensitivity with shorter scan time. However, with those approaches being strongly dependent on the input data type, it would be very difficult to perform an objective and fair comparison across the range of encoding schemes employed in this study. Moreover, the use of such complex methods for whole-brain connectivity studies is far from straightforward, because of the lack of efficient selection methods to discard the fibers arising from partial volume effects or noise.

In this work, we restrict to the analysis of DSI, QBI and DTI. However, other reconstruction schemes have been proposed; in particular, in contrast to the “model free” approaches of DSI and QBI, a number of model based methods have been proposed. Assaf and Basser have developed CHARMED [54], a composite hindered and restricted model of diffusion. Tournier et al. have proposed the CSD method [21], which estimates the fiber orientation distribution by using constrained spherical deconvolution, and which has been reported to achieve improved angular resolution and reduced orientation bias compared to QBI using water phantom data [55]. Such characteristics are likely to be due to the fact that CSD provides a direct estimate of the fiber orientation distribution (FOD, i.e. the object that is required as input to tractography algorithms) rather than an estimate of the diffusion orientation distribution function (ODF, the radial projection of the spin propagator), as provided by DSI and QBI. The latter methods estimate fiber orientations by identifying peaks in the ODF, which is inherently relatively broad.

Model based methods such as the ones mentioned above tend to build a bridge between the tensor model and model-free reconstructions, and therefore have a great potential for optimized application-driven selection of a diffusion methodology. The acquired HARDI data from this study indeed fulfill the requirements for the CSD method and may provide an interesting alternative to classical QBI that may be trimmed to reach similar or perhaps even higher sensitivity as reported here with the DSIq5 scheme. Similarly, a corrected QBI model was recently proposed to reduce the uncertainty in the orientation of the local maxima of diffusion [56], [57], and might also enhance the sensitivity of the HARDI scheme. However, due to the higher sensitivity induced by those methods, the resulting connection matrices would be in a different regime of sensitivity/specificity ratios which renders a direct comparison with DTI and DSI results difficult.

**Figure 7:**
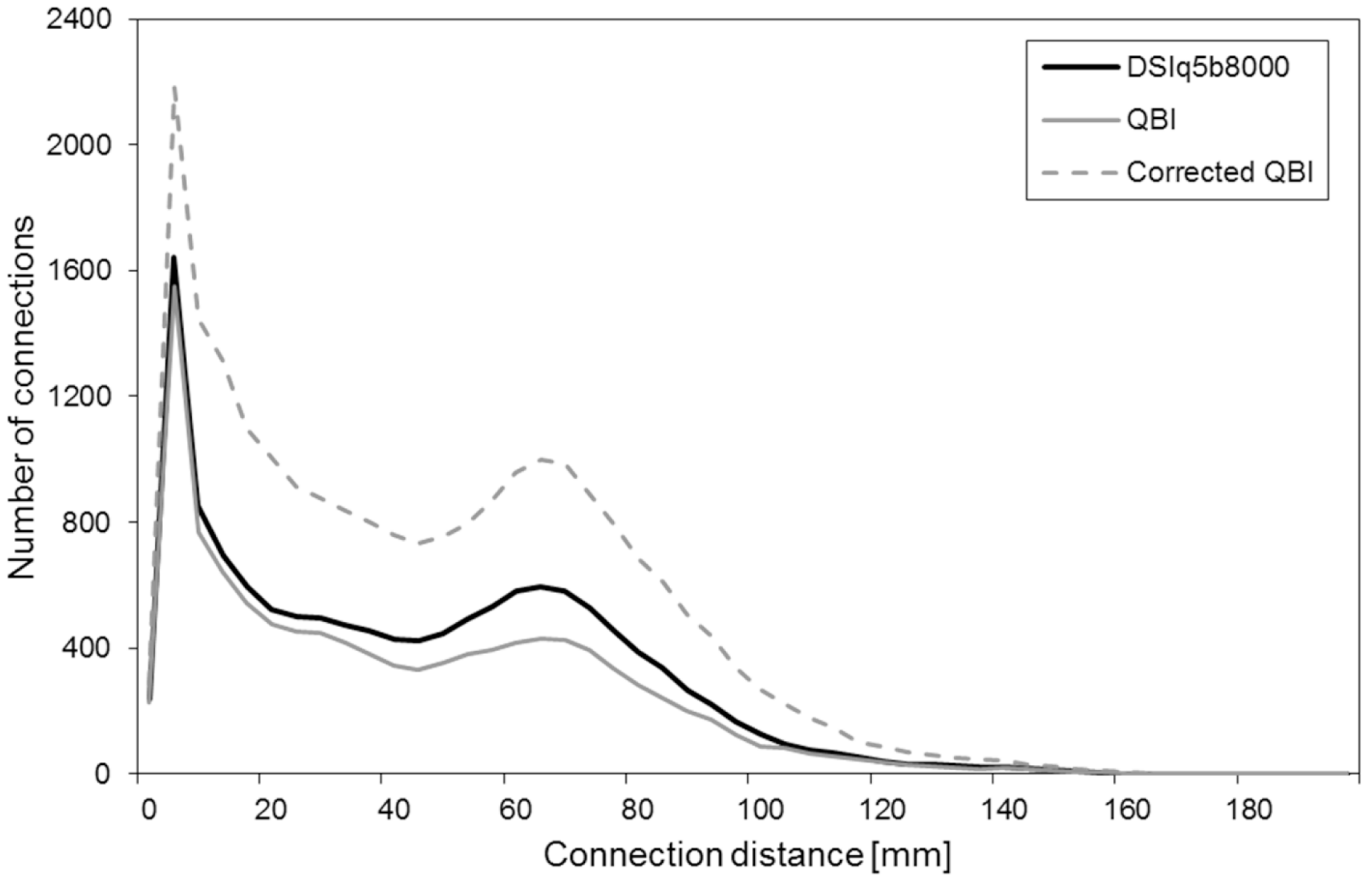
Connection distance distribution for DSIq5, QBI and the corrected QBI model. Number of connections obtained with DSIq5, QBI and the corrected QBI model as a function of the connection distance. These results are obtained by averaging over the five subjects.

This effect is demonstrated in Figure 7, which compares the number of connections obtained with DSI q5, QBI (same as Figure 4) and the corrected QBI model [56], [57]. With the corrected QBI computation, the number of connections is increased by a factor two compared to the conventional QBI reconstruction, which is in line with the theoretical consideration that the incorrect computation leads to a “smoothing” of the distribution function and thus to a reduction in fiber connections. However, in combination with the streamline algorithm we expect an amplification of false positive connections, which is apparent in the short distance connections (<40 mm). These connections are considered to be largely noisy fibers [36] and the fact that we observe a strong increase in those connections with the corrected QBI model indicates that the sensitivity and specificity in the detection of fiber connections is modulated in the updated reconstructions/tracking procedure. On the contrary, with the DTI, QBI and DSI schemes as evaluated in this investigation the number of connections in the short distance range is rather constant, indicating a good match of obtained sensitivities and specificities.

It should be noted that improvements in technology and software, including the corrected QBI and the CSD methodology, should be further explored as they may provide tremendous advantages for an optimized clinical application. However, without any ground truth information about the produced connections, any cross-comparison between reconstruction techniques without introducing normalizations between methods might be misleading due to the differences in sensitivity and specificity measures. These considerations explain why we restrict our comparison to the DTI, QBI and DSI schemes and their reconstruction and tractography within the matched processing pipeline that is expected to provide similar levels of sensitivity and specificity in fiber connections.

Finally, we should note that in this study a 32-channel head matrix coil was used and that all imaging experiments were performed with a two-fold acceleration (iPAT = 2). It is well known that the MR images exhibit a spatially inhomogeneous SNR and noise distribution when multiple channel coil arrays and parallel imaging is used for image acquisition and reconstruction [58]–[60]. In our settings, the SNR in the cortex may appear 2–3 fold higher than the SNR obtained in the center of the brain. This may raise the question whether the employed tractography algorithms are operating in a SNR limited regime. Surprisingly, our result did not show any improvements when averaging individual scans, indicating that other mechanisms such as a subtle brain and/or subject motion limit the gain in SNR. We conclude that with the given experimental setting (3T, 32-channel head coil, imaging protocol) sufficient input raw SNR is provided for stable processing and tractography analysis.

## Conclusions

In this study, we use structural connection matrices produced by tractography to assess the performance of various diffusion encoding schemes. These investigations aim at providing a framework to compare different diffusion schemes, to support a better understanding of the methodological limitations in the mapping of the human connectome. Whereas all diffusion schemes, from the classical DTI to the high angular resolution DSI, produce a biologically meaningful mapping of the human connectome, the degree of complexity of the diffusion scheme has a non-negligible influence on the sensitivity of tractography. The differences are particularly striking for non-dominant fiber populations, such as neighboring association fibers, as well as for fiber tracts that run through complex fiber crossings. For this particular type of connection, a DSI scheme with 258 encoding gradients appears most advantageous. However, depending on the application, an alternative approach that has a shorter acquisition time may be required, and indeed may be preferable due to reduced sensitivity to motion degradation of the fiber mapping.

## Supporting Information

Table S1 **Number of connections for the individual subjects. In this table, only the connections consisting in 5 fibers or more are considered.**
(DOC)

Table S2 **P-values obtained for paired t-tests performed on the number of connections, under the null hypothesis that the samples come from distributions with equal means.** In this table, only the connections consisting in 5 fibers or more are considered.
(DOC)

Table S3 **Number of connections for the individual subjects.** In this table, only the connections consisting in 20 fibers or more are considered.
(DOC)

Table S4 **P-values obtained for paired t-tests performed on the number of connections, under the null hypothesis that the samples come from distributions with equal means.** In this table, only the connections consisting in 20 fibers or more are considered.
(DOC)
