# Supplementary material for: A Connectome-Based Comparison of Diffusion MRI Schemes
Source: PLoS One. 2013 Sep 20;8(9):e75061. doi: 10.1371/journal.pone.0075061 (PMC3779224; doi:10.1371/journal.pone.0075061)
Supplement: Table S1 — Number of connections for the individual subjects. In this table, only the connections consisting in 5 fibers or more are considered. (DOC) [file pone.0075061.s001.doc]

|  | DSIq5 b8000(1) | DSIq5 b8000(2) | DSIq5 b8000(3) | DSIq5 b6400 | DSIq4 | QBI | DTI65 | DTI21 |
| --- | --- | --- | --- | --- | --- | --- | --- | --- |
| Subject 1 | 7191 | 7060 | 7044 | 7005 | 6182 | 6100 | 6019 | 5858 |
| Subject 2 | 8314 | 8177 | 8147 | 8003 | 7275 | 7003 | 6713 | 6855 |
| Subject 3 | 7755 | 8116 | 7912 | 7716 | 6909 | 6528 | 6453 | 6508 |
| Subject 4 | 7520 | 7712 | 7512 | 7381 | 6589 | 6455 | 6037 | 6170 |
| Subject 5 | 8118 | 7754 | 8103 | 7991 | 6989 | 6736 | 6592 | 6615 |
