# Supplementary material for: A Connectome-Based Comparison of Diffusion MRI Schemes
Source: PLoS One. 2013 Sep 20;8(9):e75061. doi: 10.1371/journal.pone.0075061 (PMC3779224; doi:10.1371/journal.pone.0075061)
Supplement: Table S2 — P-values obtained for paired t-tests performed on the number of connections, under the null hypothesis that the samples come from distributions with equal means. In this table, only the connections consisting in 5 fibers or more are considered. (DOC) [file pone.0075061.s002.doc]

|  | DSIq5 b8000(1) | DSIq5 b8000(2) | DSIq5 b8000(3) | DSIq5 b6400 | DSIq4 | QBI | DTI65 | DTI21 |
| --- | --- | --- | --- | --- | --- | --- | --- | --- |
| DSIq5  b8000 (1) | - | 0.91 | 0.57 | 0.02 | 3.3E-05 | 3.8E-05 | 5.6E-05 | 7.3E-06 |
| DSIq5  b8000 (2) | 0.91 | - | 0.85 | 0.27 | 2.9E-04 | 4.1E-04 | 4.1E-04 | 1.2E-04 |
| DSIq5  b8000 (3) | 0.57 | 0.85 | - | 0.01 | 3.5E-05 | 1.7E-04 | 1.0E-04 | 1.2E-05 |
| DSIq5  b6400 | 0.02 | 0.27 | 0.01 | - | 5.5E-05 | 1.2E-04 | 6.2E-05 | 8.3E-06 |
| DSIq4 | 3.3E-05 | 2.9E-04 | 3.5E-05 | 5.5E-05 | - | 0.01 | 4.2E-03 | 2.7E-05 |
| QBI | 3.8E-05 | 4.1E-04 | 1.7E-04 | 1.2E-04 | 0.01 | - | 0.04 | 0.03 |
| DTI65 | 5.6E-05 | 4.1E-04 | 1.0E-04 | 6.2E-05 | 4.2E-03 | 0.04 | - | 0.52 |
| DTI21 | 7.3E-06 | 1.2E-04 | 1.2E-05 | 8.3E-06 | 2.7E-05 | 0.03 | 0.52 | - |
