# Supplementary material for: A Connectome-Based Comparison of Diffusion MRI Schemes
Source: PLoS One. 2013 Sep 20;8(9):e75061. doi: 10.1371/journal.pone.0075061 (PMC3779224; doi:10.1371/journal.pone.0075061)
Supplement: Table S3 — Number of connections for the individual subjects. In this table, only the connections consisting in 20 fibers or more are considered. (DOC) [file pone.0075061.s003.doc]

|  | DSIq5 b8000(1) | DSIq5 b8000(2) | DSIq5 b8000(3) | DSIq5 b6400 | DSIq4 | QBI | DTI65 | DTI21 |
| --- | --- | --- | --- | --- | --- | --- | --- | --- |
| Subject 1 | 3552 | 3528 | 3560 | 3548 | 3058 | 2920 | 3174 | 3039 |
| Subject 2 | 4385 | 4280 | 4370 | 4171 | 3796 | 3653 | 3727 | 3685 |
| Subject 3 | 3964 | 4046 | 3970 | 3830 | 3652 | 3400 | 3482 | 3440 |
| Subject 4 | 3763 | 3736 | 3798 | 3763 | 3417 | 3289 | 3253 | 3234 |
| Subject 5 | 4329 | 4081 | 4188 | 4195 | 3702 | 3589 | 3626 | 3625 |
