# Supplementary material for: A Connectome-Based Comparison of Diffusion MRI Schemes
Source: PLoS One. 2013 Sep 20;8(9):e75061. doi: 10.1371/journal.pone.0075061 (PMC3779224; doi:10.1371/journal.pone.0075061)
Supplement: Table S4 — P-values obtained for paired t-tests performed on the number of connections, under the null hypothesis that the samples come from distributions with equal means. In this table, only the connections consisting in 20 fibers or more are considered. (DOC) [file pone.0075061.s004.doc]

|  | DSIq5 b8000(1) | DSIq5 b8000(2) | DSIq5 b8000(3) | DSIq5 b6400 | DSIq4 | QBI | DTI65 | DTI21 |
| --- | --- | --- | --- | --- | --- | --- | --- | --- |
| DSIq5  b8000 (1) | - | 0.30 | 0.53 | 0.08 | 1.7E-03 | 2.4E-04 | 7.8E-04 | 1.8E-04 |
| DSIq5  b8000 (2) | 0.30 | - | 0.25 | 0.60 | 1.8E-04 | 1.4E-04 | 2.2E-04 | 6.0E-05 |
| DSIq5  b8000 (3) | 0.53 | 0.25 | - | 0.13 | 5.8E-04 | 6.4E-05 | 2.5E-04 | 4.1E-05 |
| DSIq5  b6400 | 0.08 | 0.60 | 0.13 | - | 2.9E-03 | 1.5E-04 | 4.0E-04 | 7.9E-05 |
| DSIq4 | 1.7E-03 | 1.8E-04 | 5.8E-04 | 2.9E-03 | - | 3.4E-03 | 0.23 | 0.03 |
| QBI | 2.4E-04 | 1.4E-04 | 6.4E-05 | 1.5E-04 | 3.4E-03 | - | 0.16 | 0.28 |
| DTI65 | 7.8E-04 | 2.2E-04 | 2.5E-04 | 4.0E-04 | 0.23 | 0.16 | - | 0.11 |
| DTI21 | 1.8E-04 | 6.0E-05 | 4.1E-05 | 7.9E-05 | 0.03 | 0.28 | 0.11 | - |
